# Decline in ESBL Production and Carbapenem Resistance in Urinary Tract Infections among Key Bacterial Species during the COVID-19 Pandemic

**DOI:** 10.3390/antibiotics13030216

**Published:** 2024-02-26

**Authors:** Ibraheem Altamimi, Khalifa Binkhamis, Abdullah Alhumimidi, Ibrahim M. Alabdulkarim, Abdulrahman Almugren, Hadi Alhemsi, Abdulaziz Altamimi, Abeer Almazyed, Seham Elbih, Razan Alghunaim, Abdullah Altamimi

**Affiliations:** 1 College of Medicine, King Saud University, Riyadh 11461, Saudi Arabia; alhumimidia@gmail.com (A.A.); ibrahim.alabdulkarim439@gmail.com (I.M.A.); mugren10@gmail.com (A.A.); hadi.alhemsi@gmail.com (H.A.); 2 Department of Pathology, College of Medicine, King Saud University, Riyadh 11461, Saudi Arabia; kbinkhamis@ksu.edu.sa; 3 College of Medicine, King Saud Bin Abdulaziz University for Health and Sciences, Riyadh 11481, Saudi Arabia; altamimi10232@ksau-hs.edu.sa; 4 Microbiology Department, King Fahad Medical City, Riyadh 11525, Saudi Arabia; aalmazyed@kfmc.med.sa (A.A.); selbih@kfmc.med.sa (S.E.); 5 Pharmaceutical Care Division, King Faisal Specialist Hospital and Research Center, Riyadh 11211, Saudi Arabia; 6 Pediatric Emergency and Medical Toxicology, King Fahad Medical City, Riyadh 11525, Saudi Arabia; tamimi7a@gmail.com

**Keywords:** COVID-19, ESBL, carbapenem resistance, antimicrobial resistance, urinary tract infection

## Abstract

The COVID-19 pandemic has led to significant changes in healthcare practices, including increased antibiotic usage. This study aimed to investigate the impact of the pandemic on the prevalence of extended-spectrum β-lactamase (ESBL) production and carbapenem resistance among key bacterial species causing urinary tract infections (UTIs). Conducted at King Fahad Medical City in Riyadh from January 2018 to December 2022, the study analyzed urine samples from 9697 UTI patients. Patients were categorized into ‘pre-COVID-19’ and ‘during COVID-19’ groups. Bacterial isolates were identified, and antimicrobial susceptibility testing was performed following guidelines. ESBL production was detected using the Double-Disc Synergy Test. *Escherichia coli* and *Klebsiella pneumoniae* were the main pathogens. During the pandemic, ESBL production decreased in *E. coli* by 1.9% and in *K. pneumoniae* by 6.0%. Carbapenem resistance also declined, with *E. coli* displaying a 1.2% reduction and *K. pneumoniae* and *Pseudomonas aeruginosa* displaying 10.7% and 7.9% reductions, respectively. Notably, logistic regression analysis revealed that the odds of ESBL presence were 10% lower during the COVID-19 pandemic (OR 0.91; 95% CI 0.83–0.99; *p* = 0.040), and there was a significant reduction in the odds of carbapenem resistance (OR 0.43; 95% CI 0.37–0.51; *p* < 0.001). This study reveals a significant decrease in ESBL production and carbapenem resistance among UTI pathogens during the COVID-19 pandemic, hinting at the impact of modified antibiotic and healthcare approaches. It emphasizes the need for persistent antimicrobial resistance surveillance and policy adaptation to address resistance challenges, offering key directions for future public health actions.

## 1. Introduction

Urinary tract infections (UTIs) represent a significant global public health concern, affecting approximately 150 million individuals annually [1,2]. These infections, broadly categorized into uncomplicated and complicated UTIs [3,4] can lead to serious complications if left untreated, such as pyelonephritis, hypertension, renal scarring, and sepsis [5,6].

*Escherichia coli* (*E. coli*), *Klebsiella* species, and other Gram-negative bacteria are the primary pathogens responsible for UTIs [7,8,9]. These bacteria are increasingly showing resistance to a wide array of antibiotics, including penicillins, cephalosporins, and monobactams, primarily attributed to the production of extended-spectrum β-lactamase (ESBL) and carbapenem resistance [7,8,10,11]. Such resistance limits available treatment options and exacerbates morbidity and mortality [12]. Global healthcare has been significantly impacted by the emergence of these antibiotic-resistant bacteria, with the prevalence of ESBL-producing and carbapenem-resistant Enterobacterales (CRE) steadily increasing, posing a formidable challenge. This challenge has been further complicated by the COVID-19 pandemic, which has placed unprecedented strain on global healthcare resources and has led to a significant increase in the use of antibiotics.

In a local study conducted in Saudi Arabia, the prevalence of ESBL was found to be 33.4% among *E. coli* cases, indicating the severity of the issue in the region [13]. Worldwide, the situation is becoming increasingly dire. Deaths due to drug-resistant infections are projected to escalate from 700,000 to 10 million annually, with the associated cost potentially reaching USD 100 trillion globally by 2050 [14]. In places like the USA and some European countries where data are readily available, the annual cost of antimicrobial resistance (AMR) in the healthcare system has been estimated to be between $21 billion to $34 billion [15].

The COVID-19 pandemic has exerted a dramatic impact on healthcare systems globally, both in terms of financial and health outcomes. The extensive pressure on healthcare resources coupled with substantial changes in healthcare practices, including the widespread use of antibiotics, may have inadvertently influenced the rates of antibiotic resistance [16,17]. Thus, it is of paramount importance to assess the potential impact of the pandemic on these resistance rates. A recent surveillance report published by the World Health Organization (WHO) encompassing data from 22 countries showed a high level of AMR, with *E. coli* and *K. pneumoniae* identified as the two most common resistant bacteria [18]. Observations worldwide have highlighted the high dissemination of extended spectrum beta-lactamase (ESBL)-producing methicillin- and carbapenem-resistant bacteria.

The escalation of antimicrobial resistance represents a critical global public health challenge, particularly concerning ESBL-producing and carbapenem-resistant Enterobacterales (CRE) [6]. Monitoring and reporting the prevalence of ESBL-producing pathogens is essential for guiding the appropriate empirical treatment. Inappropriate antibiotic usage, a key driver of resistance, has been exacerbated by the empirical prescription of antibiotics for suspected bacterial infections and the increased use of antibiotics among COVID-19 patients [13,19]. Implementing appropriate prescription practices, optimizing antimicrobial utilization, and enhancing infection control measures may help mitigate the occurrence of drug resistance during the pandemic [20].

Against this backdrop, the present study aims to address the unexplored domain of the impact of the COVID-19 pandemic on the rates of ESBL and carbapenem resistance. This comparative study evaluates the prevalence of these multi-drug resistant organisms among UTI cases before and during the COVID-19 pandemic. The findings from this comprehensive analysis will provide valuable insights into the dynamics of antibiotic resistance trends in the era of the COVID-19 pandemic and will be crucial for guiding clinical decisions and shaping public health interventions. By providing a deeper understanding of the evolving threat of antibiotic resistance, this paper aims to inform the development of strategies that can effectively mitigate this global health challenge in the post-COVID-19 era. The study underscores the necessity of examining the impact of the COVID-19 pandemic on antibiotic resistance trends, particularly in patients with UTIs, providing critical information to guide appropriate prescription practices, optimize antimicrobial utilization, and enhance infection control measures.

## 2. Materials and Methods

### 2.1. Study Design, Setting, and Duration

The research was conducted at King Fahad Medical City (KFMC), a prominent tertiary hospital located in Riyadh, Saudi Arabia. This hospital serves as a referral center for patients from local health centers, private hospitals, and clinics. The Institutional Review Board of KFMC approved this study (IRB 23333) and waived the requirement of obtaining informed consent from the participants owing to the retrospective nature of the study. The retrospective, cross-sectional study spanned from 1 January 2018 to 31 December 2022, and it aimed to comprehensively examine all positive urine cultures submitted for culture and sensitivity testing during this period. The timeline for categorizing the COVID-19 period was established based on the date of the first identified case [20]. Any urine culture identified before 3 March 2020 was classified as ‘pre-COVID-19’, reflecting a period devoid of the disease’s impact. This demarcation was crucial for analyzing measures and countermeasures implemented in response to the pandemic. Subsequently, any urine culture identified on or after 3 March 2020 was classified as ‘during COVID-19’, signifying the onset and continuation of the pandemic [21]. This temporal classification was essential for the accurate assessment of the pandemic’s progression and the effectiveness of the implemented strategies.

### 2.2. Inclusion and Exclusion Criteria

The study population was carefully selected based on precise inclusion criteria designed to identify patients with clinical suspicion of urinary tract infections (UTIs). These inclusion criteria targeted individuals displaying symptoms indicative of UTIs, including fever exceeding 38 °C, dysuria, hesitancy, frequency, urgency, low-volume voids, or discomfort in the lower abdomen. For infants and children, a urinary tract infection is identified when a single pathogen exceeds 10 × 10^5^ colony-forming units per milliliter (CFU/mL) in a properly collected urine sample (such as from suprapubic aspiration, transurethral catheterization, or mid-stream urine) in children who present with fever or other symptoms related to the urinary system [17,22,23].

Furthermore, the study specifically focused on patients whose urine cultures yielded positive results for *E. coli*, *K. pneumoniae*, and *P. aeruginosa*. To ensure the robust characterization of infections, only urine specimens with a substantial microbial load exceeding 10^5^ colony-forming units per milliliter (CFU/mL) were included [24]. This stringent inclusion criterion was established to facilitate a comprehensive and accurate analysis of UTI prevalence and resistance patterns within the defined study population.

To ensure the integrity and validity of the study’s findings, meticulous exclusion criteria were established. Urine samples containing multiple types of bacteria were excluded to eliminate ambiguity in the results. Samples collected over three days post-hospital admission were also omitted to minimize the impact of nosocomial infections. Additionally, samples from patients with chronic renal failure or severe birth defects as well as those collected using pediatric bags were excluded to prevent contamination and confounding factors [13,17]. The study rigorously adhered to European guidelines for the registration, documentation, and transportation of samples to the microbiological laboratory. Strict protocols for sample handling and storage were implemented, ensuring sample integrity even in cases where immediate transportation was not feasible. Furthermore, to maintain sample quality, they were refrigerated at controlled temperatures between 4 °C and 6 °C [13,25]. These comprehensive criteria were thoughtfully crafted to guarantee the study’s reliability, accuracy, and commitment to scientific rigor and validity.

### 2.3. Methodology for the Cultivation and Analysis of Urine Samples: Adhering to WHO Guidelines and Utilizing Semi-Quantitative Techniques

Culturing of urine samples was performed using a semiquantitative approach in line with the protocols recommended by the World Health Organization (WHO), employing CLED and blood agar [26]. In this process, each urine sample, precisely 1 mL, was spread on plates containing cystine-lactose-electrolyte-deficient (CLED) and blood agar (sourced from Hardy Diagnostics, Santa Maria, CA, USA) [27]. These plates were subsequently subjected to aerobic incubation at a temperature of 37 °C for 24 h, facilitated by the use of a calibrated wire loop. To characterize and identify the microbial isolates, a combination of biochemical testing and analysis of cultural characteristics was utilized, employing systems such as the BD Phoenix (provided by BD-Canada, Mississauga, ON, Canada) and the API ID (from bioMerieux UK Ltd., Basingstoke, UK) [28].

### 2.4. Application of the BD Phoenix System in Bacterial Isolate Identification

The BD Phoenix system’s NMIC/ID-4 panel was utilized for the analysis of 9697 bacterial isolates. A stock solution with a McFarland standard of 0.5 was prepared using 4.5 mL of Phoenix ID broth and a Sensi Titre™ nephelometer (Thermo Fisher Scientific, Waltham, MA, USA) [29]. This bacterial identification solution was then allocated to the appropriate ID area on the Phoenix panel. Subsequently, 50 µL of the bacterial solution was dispensed into each of the chemical reactivity wells present on the panel. After sealing the panel and reading the code, it was inserted into the Phoenix machine for processing. The identification results obtained from the Phoenix system for these bacterial isolates were then cross-referenced with those from the conventional API system, enabling the determination of identification accuracy at both the genus and the species levels [30].

### 2.5. Antimicrobial Susceptibility Testing of E. coli, Pseudomonas, and Klebsiella Strains Using the Kirby–Bauer Method for Carbapenem Resistance Identification

The in vitro antimicrobial susceptibility testing of isolated *E. coli*, *P. aeruginosa*, and *K. pneumoniae* strains was conducted following the Clinical Laboratory Standard Institute (CLSI) guidelines, version 6.0. All identified bacterial isolates were subjected to in vitro investigation of their antimicrobial sensitivity using the agar disc diffusion technique (Hardy Diagnostics, Santa Maria, CA, USA) [31]. The isolates were categorized as susceptible (S), intermediate (I), or resistant (R) to the tested antibiotics, and multidrug-resistant classification was applied as necessary.

### 2.6. Application of the Double-Disc Synergy Test (DDST) for ESBL Detection

ESBL detection in bacterial pathogens (*E. coli* ATCC 25922, *K. pneumoniae* ATCC 700603) was performed using the DDST method. The test utilized ceftazidime (30 μg), cefotaxime (30 μg), cefpodoxime (30 μg), and amoxicillin/clavulanic acid (amoxicillin 20 μg + clavulanic acid 10 μg). On Muller–Hinton agar (MHA) plates, the relevant discs were placed at a specific distance and, following overnight incubation at 37 °C, an increase in the inhibition zone of more than 5 mm according to EUCAST guidelines indicated a positive ESBL finding [32].

### 2.7. Statistical Analysis

Data analysis was performed using commercially available SPSS (IBM Corporation, New York, NY, USA, Version 25). After collecting data on dedicated Microsoft Excel sheets, they were reorganized to obtain the data relevant to the target bacteria of the study and subsequently exported to SPSS and coded for analysis. Categorical variables were presented as frequencies and percentages. Age was the only continuous variable and was presented as mean values with standard deviation after checking for normal distribution through the use of histogram plots (visually) and the Kolmogorov–Smirnov test for normality (statistically).

The chi-square test was used to check for statistically significant associations between categorical variables, while the independent samples *t*-test was utilized to find statistically significant differences in the mean ages grouped by either ESBL presence or carbapenem resistance. A binary logistic regression analysis was performed to uncover the association between the presence of a predictor variable and the likelihood of ESBL presence or carbapenem resistance. Finally, a multilogistic regression analysis was performed to determine the likelihood of carbapenem resistance among each of the three bacteria relative to each other. This study utilized multivariate-adjusted odds ratios (OR) with 95% confidence intervals to evaluate the relationship between the dependent variables and the participant characteristics. A *p*-value of less than 0.05 was regarded as being statistically significant for all analyses.

## 3. Results

### 3.1. ESBL Production

Out of the 14,666 cultures studied, 8631 (58.8%) were urine samples that were included in the study. Of the patients with urine samples, under 50% of the sample consisted of patients before the COVID-19 pandemic, while an approximate majority were participants included during the pandemic. Analysis of the demographic distribution of the participants revealed that approximately 72% of the cultures were obtained from female subjects (n = 6214), while the remaining 28% were from male subjects (n = 2417). Regarding age-wise distribution, approximately 20% of the sample were individuals under the age of 18 years (n = 1726), while the remainder were aged 18 and above (m = 6905). In addition, a vast majority of the cultures were derived from inpatient participants (approximately 96%). *E. coli* was identified in 6270 (72.7%) of the study sample, while *K. Pneumoniae* was isolated in 2234 (25.8%) samples. A small proportion consisting of only 127 (1.5%) samples contained other types of bacteria. In terms of antibiotic resistance, extended spectrum beta-lactamase (ESBL) was detected in 31% of the samples, whereas the remaining 69% of the samples did not exhibit the presence of ESBL. The minimum age of the participants was 1 day old, and the maximum age was 106 years.

**Figure 1:**
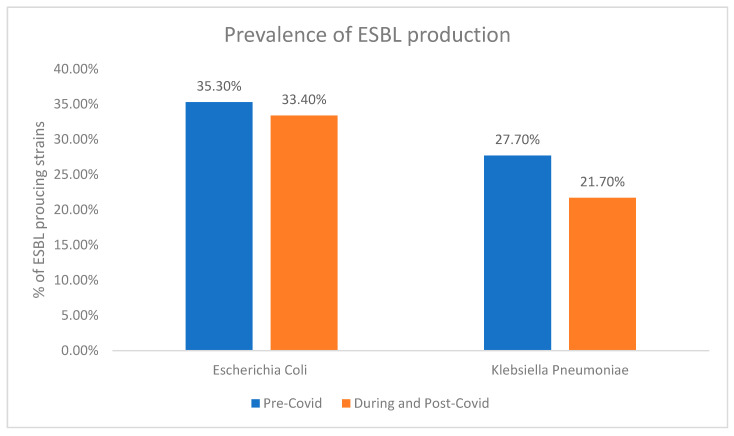
Comparison of ESBL prevalence among *E. coli* and *K. pneumoniae* strains pre-COVID and during COVID.

Analysis of the prevalences of ESBL production in each bacterium when grouped based on being prior to versus during the COVID-19 pandemic revealed a decline in both bacterial species amidst the COVID-19 pandemic. In particular, *E. coli* demonstrated a 1.9% reduction in ESBL production, whereas *K. pneumoniae* demonstrated a decrease of 6.0% in the same parameter (Figure 1).

In terms of pre-COVID19 analysis of ESBL production for *E. coli*, the prevalence of ESBL production was 35.3%. When stratified by gender, ESBL production was noted to be higher in males with statistical significance (*p* = 0.000). Similarly, stratification and analysis based on patient population revealed a higher prevalence of ESBL production among outpatients (55.6%) when compared to inpatients (n = 34.8%) with statistical significance (*p* = 0.001). Finally, a statistically significant difference was also found for mean age and ESBL production, in that mean age was higher among participants with ESBL producing *E. coli* (*p* = 0.000) (Table 1). Meanwhile, analysis of ESBL production for *E. coli* during COVID-19 returned similar findings, with a higher prevalence of ESBL production being noted for males as compared to females (44.1% vs. 30.2%, *p* = 0.000) and for outpatients as compared to inpatients (55.6% vs. 34.8%, *p* = 0.001) and a higher mean age for participants with ESBL production (*p* = 0.000) (Table 2).

On the other hand, pre-COVID analysis of ESBL production in *K. pneumoniae* revealed that, while the prevalence of ESBL production was higher in males when compared to females, the result was not statistically significant. Similarly, no statistically significant differences were found between the prevalences of ESBL production in inpatients when compared with outpatients. Unlike ESBL production in *E. coli*, however, the mean age was noted to be lower in patients with ESBL producing *K. pneumoniae* strains with statistical significance (*p* = 0.028) (Table 3). In terms of analysis of ESBL production for *K. pneumoniae* during COVID, no statistically significant differences were found based on gender, age, or patient type, although males were once again noted to have a higher prevalence of ESBL production (Table 4).

**Figure 2:**
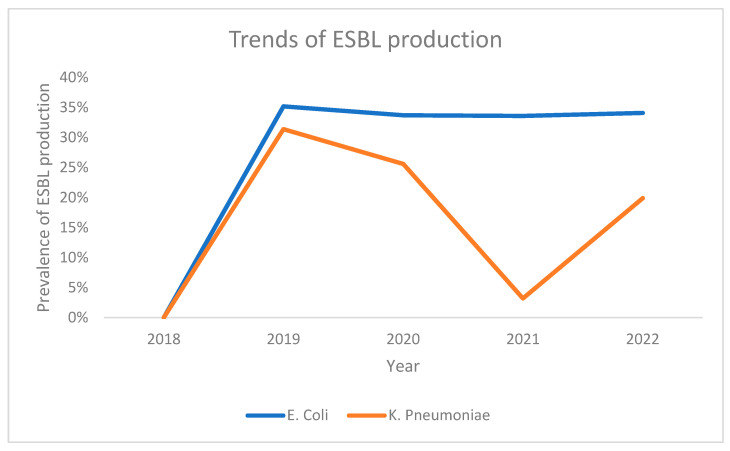
Trends of ESBL production among *E. coli* and *K. pneumoniae* across the study period.

Analyzing trends of ESBL production for both bacteria based on the year of sample collection revealed that the lowest prevalence was seen in the year 2018 for both *E. coli* and *K. pneumoniae*. Both bacteria demonstrated a sharp rise in ESBL production for the following year (2019), with the *E. coli* trend line demonstrating somewhat of a plateau in the subsequent years. Meanwhile, the trend for *K. pneumoniae* was more variable, decreasing in both 2020 and 2021 but demonstrating a sharp rise in ESBL production in the year 2022 (Figure 2).

**Figure 3:**
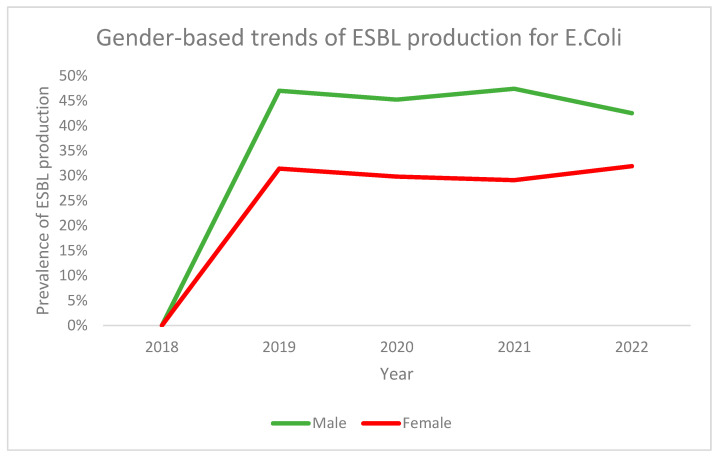
Gender-based trends of ESBL production for *E. coli* across the study period.

**Figure 4:**
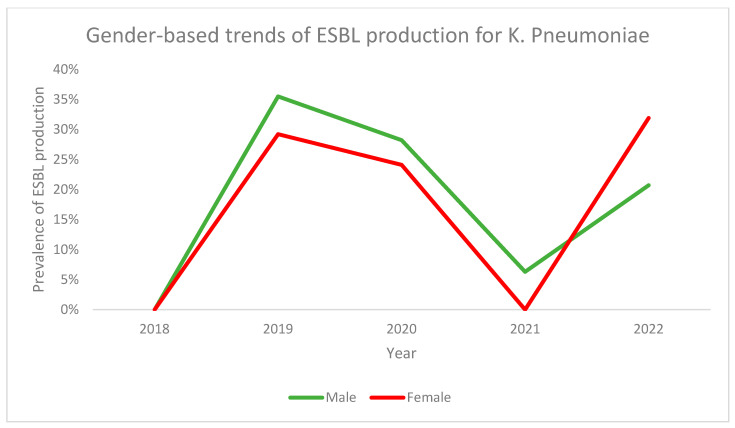
Gender-based trends of ESBL production for *K. pneumoniae* across the study period.

On the other hand, analysis of gender-based trends for ESBL production in *E. coli* revealed that, while males had a higher prevalence throughout the time period, the trends were similar in both genders. Both had the lowest prevalence in 2018 and demonstrated a sharp increase in 2019 followed by a gradual decline or maintenance (Figure 3). Both genders saw their lowest prevalence in 2018 followed by a sharp increase in 2019. Subsequently, the prevalence decreased until 2021 and then increased again in 2022 (Figure 4).

A binary logistic regression analysis was conducted to ascertain the predictors of extended spectrum beta-lactamase (ESBL) presence. The independent variables included bacteria type, gender, age, patient type (inpatients or outpatients), and pre-COVID/during COVID. The analysis was performed with a significance level of 0.05. The model demonstrated significance (*p* < 0.001) and accurately classified 73% of cases. However, only 2.7% of the variation in ESBL presence could be accounted for by the model.

All variables were significant predictors within the model. The odds of ESBL production were 1.51 times higher for males compared to females. For *E. coli*, the odds of ESBL production were 1.60 times higher than for *Klebsiella* and other bacterial species. Holding all other factors constant, a unit increase in age was anticipated to raise the odds of ESBL presence by one. However, the odds ratio of one indicates that the relationship between ESBL presence and age is not significant. Inpatients exhibited 0.74 times lower odds of ESBL production compared to outpatients, and samples collected during COVID demonstrated 10% lower odds of ESBL production (Table 5).

### 3.2. Carbapenem (CP) Resistance

A total of 9697 participants had their urine samples for analysis, the majority of which revealed *E. coli* growth (64.7%) followed by *Klebsiella pneumoniae* growth (22.9%) and *Pseudomonas aeruginosa* growth (11.0%). Approximately 20% of the participants were aged under 18 years old (n = 1920), while the remainder were aged 18 years old or above (n = 7777). A small proportion of the sample (1.4%) revealed growth of other bacteria. Out of the total number of participants, approximately 38% had their samples collected prior to COVID-19, while the remaining 62% had their samples collected during COVID-19. Females made up the majority of the study sample gender-wise (approximately 69%), and only 273 (2.8%) of the sample were outpatients.

**Figure 5:**
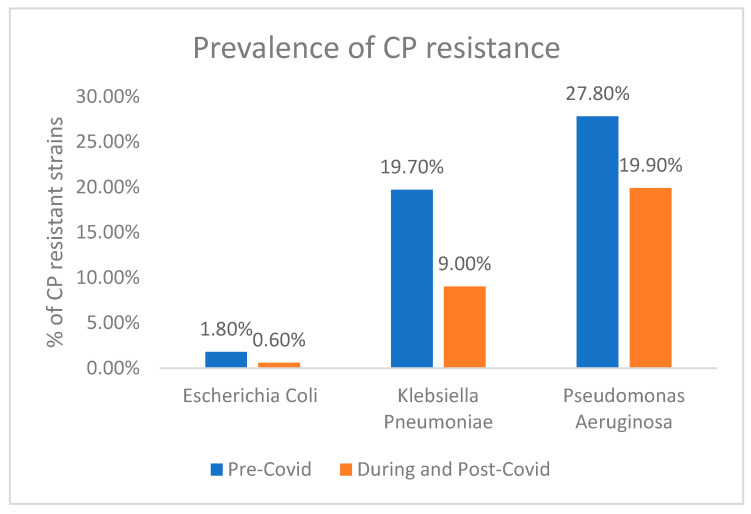
Comparison of prevalence of CP resistance among *E. coli*, *P. aeruginosa*, and *K. pneumoniae* strains pre- and during COVID.

Analysis of the prevalence of carbapenem resistance (CPR) in each bacterium when grouped based on being prior to versus during the COVID-19 pandemic revealed a decline in both bacterial species amidst the COVID-19 pandemic. In particular, *E. coli* demonstrated a 1.2% reduction in CPR, *K. pneumoniae* demonstrated a decrease of 10.7%, and *P. aeruginosa* demonstrated a decrease of 7.9% in the same parameter (Figure 5).

Pre-COVID analysis of CPR for *E. coli* revealed that the prevalence was only slightly higher in males when compared to females, with the overall result not being statistically significant. No outpatient demonstrated CPR, and no statistically significant differences were found in CPR prevalence when stratified by patient population. Age-based analysis between patients CPR and carbapenem sensitive (CPS) strains revealed that, while the mean age was noted to be higher among patients with CPR, the overall result was not statistically significant (Table 6).

For CPR analysis during COVID-19, the prevalence of CPR was found to be comparable in both males and females and among inpatients and outpatients. While the mean age for patients with CPS strains was higher compared to CPR strains, the analysis was once again not statistically significant (Table 7).

In terms of pre-COVID CPR analysis for *K. pneumoniae*, the prevalence was noted to be higher in males when compared to females (23.5% vs. 17.6%) with statistical significance (*p* = 0.027). Meanwhile, while the prevalence of CPR was higher for outpatients (30.6%) compared to inpatients (19.3%), the differences were not statistically significant. Finally, analysis of the mean age between patients with CPR strains versus those with CPS strains revealed a higher mean age for those with CPR strains with statistical significance (*p* = 0.000) (Table 8).

On the other hand, CPR analysis for *K. pneumoniae* during COVID-19 revealed similar findings, with a higher prevalence seen in males when compared to females (13.4% vs. 6.6%, *p* = 0.000) and a higher mean age for patients with CPR strains when compared to patients with CPS strains (*p* = 0.005). Similar to the pre-COVID analysis, the prevalence of CPR was noted to be higher among outpatients, but the overall result was not statistically significant (Table 9).

Pre-COVID CPR analysis for *P. aeruginosa* stratified by gender, age, and patient classification revealed no statistically significant differences between any of the tested variables (Table 10). The prevalences of CPR were comparable among both genders and both patient populations, albeit there was a slightly higher prevalence among inpatient participants.

Similarly, analysis of CPR for *P. aeruginosa* during COVID-19 revealed that the differences in gender-based and classification-based CPR prevalences were not statistically significant. In addition, while the mean age was noted to be slightly higher among patients with CPR, the result was once again not statistically significant (Table 11).

**Figure 6:**
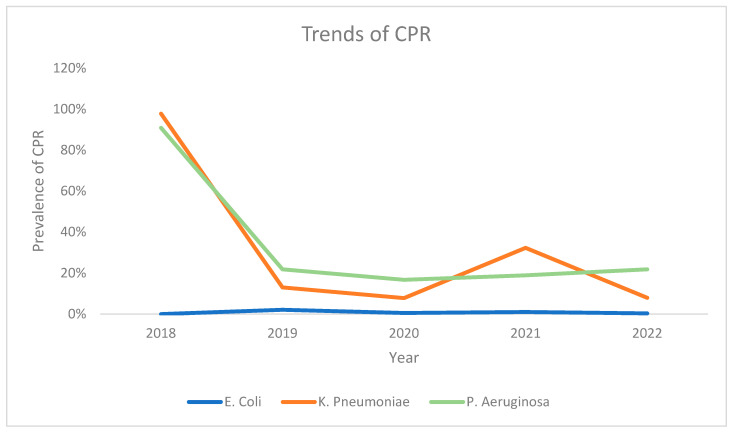
Trends of CP resistance among *E. coli*, *P. aeruginosa*, and *K. pneumoniae* across the study period.

Analysis of the carbapenem resistance trends for each of the three bacteria based on the year of sample collection revealed that *E. coli* had the lowest prevalence of CPR every year. Meanwhile, the starting CPR prevalence for *K. pneumoniae* was higher than *P. aeruginosa* in 2018 and in 2021. For the other years (2019, 2020, and 2022), *P. aeruginosa* had a higher prevalence of CPR when compared with *K. pneumoniae*, with both bacteria demonstrating somewhat of a decreasing trend (the exception being *K. pneumoniae* in 2021) from a high initial CPR prevalence in 2018 (Figure 6).

**Figure 7:**
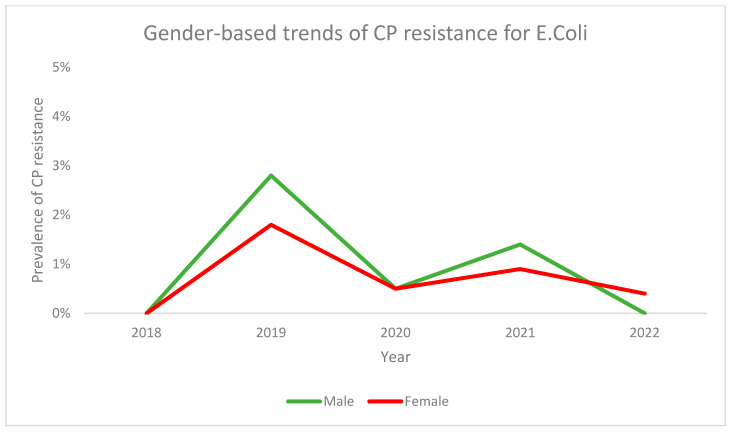
Gender-based trends for CP resistance in for *E. coli* across the study period.

Gender-based analysis for prevalence of CPR among patients with *E. coli* revealed similar trends for both males and females, with higher overall prevalence being seen in males. Both genders saw an increase in prevalence from 2018 to 2019 followed by a decrease in 2020, then a short rise in 2021 followed by another decrease in 2022. The prevalence in females was lower than males at all time points except in 2022 (Figure 7).

**Figure 8:**
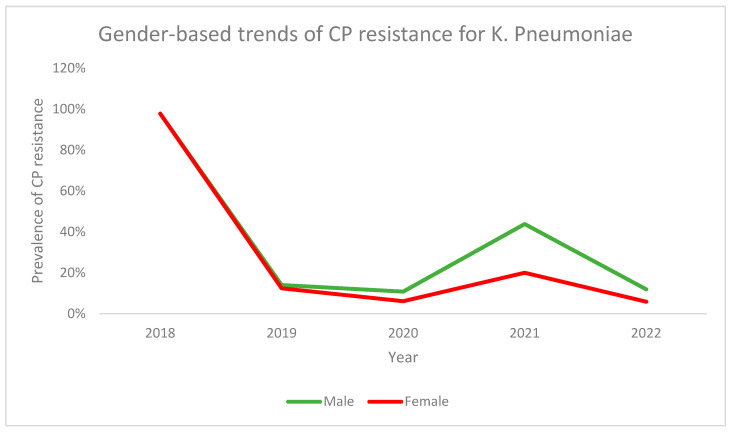
Gender-based trends for CP resistance among *K. pneumoniae* across the study period.

Similarly, gender-based analysis for prevalence of CPR among patients with *K. pneumoniae* also revealed similar trends for both males and females, with males having a higher overall prevalence. The CPR trend in both genders demonstrated a decrease from a high starting point in 2018 to 2019. The prevalence of CPR was noted to increase in 2021 among both genders, although a sharper rise was noted for males. This was followed by a decrease in CPR prevalence in 2022 for both genders, although, once again, the decline was sharper in males (Figure 8).

**Figure 9:**
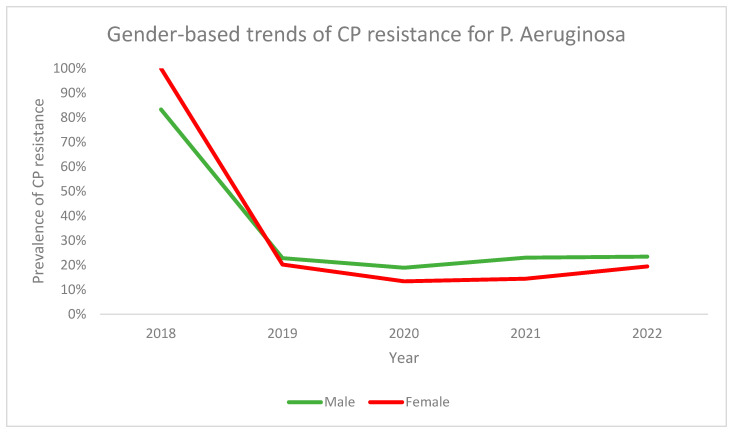
Gender-based trends for CP resistance in *P. aeruginosa* across the study period.

Finally, gender-based trends for CPR prevalence in *P. aeruginosa* revealed that females started off with a higher CPR prevalence in 2018 as compared to males, and both genders saw a decline in CPR prevalence from 2018 to 2019. Across all the following years, the overall prevalence of CPR was lower in females as compared to males with the trend lines demonstrating a similar plateau-like pattern (Figure 9).

A logistic regression analysis was performed to predict carbapenem resistance using the following predictor variables: type of bacteria, gender, age, patient type, and the period before/during the COVID-19 pandemic. The model was found to be statistically significant (*p* < 0.001) and accurately classified 96% of the cases. Approximately 21% of the variance in carbapenem resistance can be accounted for by the model.

All predictor variables were statistically significant except patient type. The odds of *E. coli* being associated with carbapenem resistance were 94% lower compared to *K. pneumoniae* and 97% lower compared to *P. aeruginosa*. The odds of carbapenem resistance were noted to be 1.93 times higher in *P. aeruginosa* when compared with *K. pneumoniae* (Table 12).

Concerning gender, males exhibited 2.5 times higher odds of carbapenem resistance compared to females. Additionally, a one unit increase in age was associated with a corresponding increase in the odds of carbapenem resistance by a factor of one. Lastly, the odds of carbapenem resistance during the COVID-19 pandemic were approximately 60% lower than those observed before the pandemic (Table 13).

## 4. Discussion

In discussing the trends of extended spectrum β-lactamase (ESBL) and carbapenem resistance, our study presents a distinct contrast to earlier research, especially in the context of the COVID-19 pandemic. Previous studies such as Bradford [33] reported a growing prevalence of ESBL-producing bacteria, particularly in *E. coli* and *K. pneumoniae*. The WHO’s 2018 [34] survey echoed this, noting alarmingly high rates of ESBL production in these bacteria, especially in healthcare settings. Karanika et al. [35] further emphasized the health and economic burdens posed by these bacteria.

In contrast, our study during the pandemic period observed a decrease in ESBL production: *E. coli* exhibited a 1.9% reduction, and *K. pneumoniae* showed a 6.0% decrease. This is in stark contrast to findings from Riyadh, where ESBL production in *E. coli* was reported at 32% and 33%, and in Klebsiella, it varied between 37.75% and a significant 63% [13]. In a French study, a notable decrease in ESBL *E. coli* prevalence was observed from a pre-March 2020 rate of 9.3% (ranging from 6.5–10.5%) to 8.3% (ranging from 7.2–9.1%) post-May 2020 with a statistically significant *p*-value (*p* < 0.001). The rate of reduction in ESBL *E.coli* accelerated significantly after May 2020, moving from a monthly decrease of −0.04% to −0.22% with a significant *p*-value (*p* < 0.001), suggesting an impactful change during the pandemic [36]. The observation from Indonesia, which highlighted a significant decrease in ESBL production among *E. coli* from 50% in group A to 20.9% in group B with a *p*-value of 0.000, adds substantial evidence to the notion that the COVID-19 pandemic may have profoundly influenced the dynamics of antimicrobial resistance (AMR) in certain regions in various demographic groups and healthcare settings. This provides a concrete example that contrasts with the global trends of rising antibiotic resistance [37].

Our findings indicating a reduction to below 2% in *E. coli* and approximately 35% in *K. pneumoniae* suggest a possible shift in resistance patterns potentially due to changes in antibiotic usage, infection control measures, or other pandemic-related factors. This divergence is noteworthy, highlighting the unique impact of the pandemic on bacterial resistance trends and underscoring the need for ongoing surveillance and research in this area.

In our study, a significant decline in carbapenem resistance was observed across three bacterial species during the COVID-19 pandemic, with *E. coli* dropping by 1.2%, *K. pneumoniae* by 10.7%, and *P. aeruginosa* by 7.9% This contrasts with findings from other studies, such as Nordmann et al. [38], Tacconelli et al. [39], Adegoke et al. [40], and Mares et al. [41], which reported a global rise in carbapenem resistance, particularly in *K. pneumoniae* and *P. aeruginosa*. The research by Mares et al. revealed significant changes in antibiotic resistance among *Pseudomonas* spp., particularly concerning carbapenems. There was a notable increase in resistance rates for imipenem, escalating from 18.18% to 67.74%, and for meropenem, escalating from 18.18% to 64.51% [41]. Another local study in Jeddah showed an increase in resistance among Enterobacteriaceae from 8% to 13% [42]. In contrast to previous studies, a Romanian study highlighted a stable sensitivity to carbapenems among *E. coli*, *K. pneumoniae*, and *P. aeruginosa* over the observation period of 2018–2022 [43]. The decreases observed in our study, notably in *E. coli* and *K. pneumoniae*, sharply contrast with these rising global trends, suggesting that the pandemic may have influenced these patterns, possibly due to changes in antibiotic prescribing or infection control measures. This divergence highlights the dynamic nature of antibiotic resistance and underlines the importance of ongoing research and surveillance to understand whether these pandemic-related shifts represent a temporary change or a more lasting trend, which is critical for developing strategies against carbapenem-resistant organisms in healthcare settings.

Acknowledging this point, it is important to clarify that the referenced studies, including those by Nordmann et al. [38] and Tacconelli et al. [39], and the research conducted in Jeddah did not specifically compare carbapenem resistance prevalence before and during the COVID-19 pandemic. Instead, they reported on the prevalence of carbapenem resistance at specific time points, highlighting an overall increasing trend in resistance, particularly in strains such as *K. pneumoniae* and within the Enterobacteriaceae family. This context emphasizes the uniqueness of our study, which specifically examines the shift in carbapenem resistance trends during the COVID-19 pandemic, offering a comparative perspective that is not directly addressed in these other studies.

### Strengths and Limitations

The study under discussion stands out as the first worldwide to compare extended spectrum beta-lactamase (ESBL) and carbapenem resistance in urine samples before and during the COVID-19 pandemic. Its robust sample size, encompassing 9697 urine samples, adds considerable weight to its findings. Additionally, the study aligns with the World Health Organization’s Global Action Plan, which emphasizes the importance of monitoring antimicrobial resistance (AMR) as a critical step in combating its spread. This aspect of the study not only enhances its relevance but also contributes to a broader understanding of AMR trends in the context of a global health crisis.

However, the study is not without its limitations. The retrospective nature of the research introduces inherent constraints typical of this approach, such as potential biases in data collection and interpretation. The patient population, drawn from diverse regions in Saudi Arabia and admitted to the King Fahad Medical City (KFMC), may not fully represent the broader demographic, thus limiting the generalizability of the findings. Additionally, there is a possibility that certain cases were missed, particularly those where patients received treatment without a preceding urine culture or had prior antibiotic administration. It is also notable that our analysis did not include *P. aeruginosa* in the examination of ESBL production due to incomplete data. This omission is particularly significant, as *P. aeruginosa* is a crucial bacterium in ESBL production. These gaps highlight areas where the study’s methodology could be improved for more accurate results.

Looking ahead, it is evident that future research in this area needs to adopt a more expansive approach. Studies that incorporate larger and more diverse patient populations coupled with longitudinal analyses would provide a more comprehensive view of susceptibility patterns. Such research would not only illuminate variations in antimicrobial resistance before and during the pandemic but also offer valuable insights into the evolving nature of AMR in response to global health emergencies. This future direction is essential for a deeper and more accurate understanding of AMR trends, particularly in the context of unprecedented health challenges like the COVID-19 pandemic.

## 5. Conclusions

In conclusion, the study provides pivotal insights into the dynamics of antimicrobial resistance amid the COVID-19 pandemic, highlighting a significant decrease in ESBL production among key bacterial species like *E. coli* and *K. pneumoniae* and a notable reduction in carbapenem resistance across *E. coli*, *K. pneumoniae*, and *P. aeruginosa*. These trends, which might be attributed to changes in antibiotic usage and healthcare practices during the pandemic, underline the necessity for ongoing surveillance and research in antimicrobial resistance. The findings emphasize the importance of adapting antibiotic stewardship programs and healthcare policies to effectively respond to evolving resistance patterns, especially in the context of global health emergencies.

## Figures and Tables

**Table 1:** Pre-COVID-19 analysis of extended spectrum beta-lactamase (ESBL) production stratified by gender, age, and patient classification for *E. coli* (n = 2267).

Variable	ESBL Producing Strains (n = 801)	Non-ESBL Producing Strains (n = 1466)	*p*-Value
**Gender** - Male - Female	**(n, %)** 262 (47.1) 539 (31.5)	(**n, %)** 294 (52.9) 1172 (68.5)	0.000
**Age in years (Mean ± S.D)**	44.3 ± 26.5	40.3 ± 24.3	0.000
**Patient type** - Inpatient - Outpatient	**(n, %)** 766 (34.8) 35 (55.6)	**(n, %)** 1438 (65.2) 28 (44.4)	0.001

**Table 2:** During-COVID-19 analysis of extended spectrum beta-lactamase (ESBL) production stratified by gender, age, and patient classification for *E. coli* (n = 4033).

Variable	ESBL Producing Strains (n = 1349)	Non-ESBL Producing Strains (n = 2680)	*p*-Value
**Gender** - Male - Female	**(n, %)** 418 (44.1) 931 (30.2)	**(n, %)** 529 (55.9) 2151 (69.8)	0.000
**Age in years (Mean ± S.D)**	47.8 ± 26.0	42.2 ± 25.0	0.006
**Patient type** - Inpatient - Outpatient	**(n, %)** 1306 (33.2) 43 (43.9)	**(n, %)** 2629 (66.8) 55 (56.1)	0.027

**Table 3:** Pre-COVID-19 analysis of extended spectrum beta-lactamase (ESBL) production stratified by gender, age, and patient classification for *K. pneumoniae* (n = 1006).

Variable	ESBL Producing Strains (n = 279)	Non-ESBL Producing Strains (n = 727)	*p*-Value
**Gender** - Male - Female	**(n, %)** 110 (30.2) 169 (26.3)	**(n, %)** 254 (69.8) 473 (73.7)	0.185
**Age in years (Mean ± S.D)**	41.6 ± 28.3	45.9 ± 27.9	0.028
**Patient type** - Inpatient - Outpatient	**(n, %)** 273 (28.1) 6 (17.6)	**(n, %)** 699 (71.9) 28 (82.4)	0.181

**Table 4:** During-COVID-19 analysis of extended spectrum beta-lactamase (ESBL) production stratified by gender, age, and patient classification for *K. pneumoniae* during (n = 1228).

Variable	ESBL Producing Strains (n = 266)	Non-ESBL Producing Strains (n = 962)	*p*-Value
**Gender** - Male - Female	**(n, %)** 97 (22.8) 169 (21.0)	**(n, %)** 328 (77.2) 634 (79.0)	0.472
**Age in years (Mean ± S.D)**	45.9 ± 28.5	48.5 ± 25.2	0.149
**Patient type** - Inpatient - Outpatient	**(n, %)** 256 (21.7) 10 (21.7)	**(n, %)** 926 (78.3) 36 (78.3)	0.990

**Table 5:** Results of logistic regression analysis for predicting extended spectrum beta-lactamase (ESBL) presence.

Variable	OR (95% CI)	*p*-Value
*E. coli*	1.60 (1.44–1.79)	0.000
Male	1.51 (1.37–1.67)	0.000
Age	1.00 (1.00–1.04)	0.000
Inpatients	0.74 (0.57–0.96)	0.024
During COVID	0.91 (0.83–0.99)	0.040

**Table 6:** Pre-COVID-19 analysis of carbapenem resistance stratified by gender, age, and patient classification for *E. coli* (n = 2267).

Variable	CP Resistant Strains (n = 41)	CP Sensitive Strains (n = 2226)	*p*-Value
**Gender** - Male - Female	**(n, %)** 14 (2.6) 27 (1.6)	**(n, %)** 534 (97.4) 1682 (98.4)	0.177
**Age in years (Mean ± S.D)**	47.2 ± 26.9	41.7 ± 25.5	0.179
**Patient type** - Inpatient - Outpatient	**(n, %)** 41 (1.9) 0	**(n, %)** 2173 (98.1) 53 (100)	0.327

**Table 7:** During COVID-19 analysis of carbapenem resistance stratified by gender, age, and patient classification for *E. coli* (n = 4033).

Variable	CP Resistant Strains (n = 24)	CP Sensitive Strains (n = 4009)	*p*-Value
**Gender** - Male - Female	**(n, %)** 6 (0.6) 18 (0.6)	**(n, %)** 942 (99.4) 3067 (99.4)	0.953
**Age in years (Mean ± S.D)**	41.3 ± 23.1	44.1 ± 25.5	0.588
**Patient type** - Inpatient - Outpatient	**(n, %)** 24 (0.6) 0	**(n, %)** 3911 (99.4) 98 (100)	0.429

**Table 8:** Pre-COVID-19 analysis of carbapenem resistance stratified by gender, age, and patient classification for *K. Pneumoniae* (n = 1006).

Variable	CP Resistant Strains (n = 198)	CP Sensitive Strains (n = 808)	*p*-Value
**Gender** - Male - Female	**(n, %)** 84 (23.5) 114 (17.6)	**(n, %)** 273 (76.5) 535 (82.4)	0.027
**Age in years (Mean ± S.D)**	53.3 ± 27.0	42.7 ± 27.9	0.000
**Patient type** - Inpatient - Outpatient	**(n, %)** 187 (19.3) 11 (30.6)	**(n, %)** 782 (80.7) 26 (69.4)	0.096

**Table 9:** During COVID-19 analysis of carbapenem resistance stratified by gender, age, and patient classification for *K. pneumoniae* (n = 1228).

Variable	CP Resistant Strains (n = 110)	CP Sensitive Strains (n = 1117)	*p*-Value
**Gender** - Male - Female	**(n, %)** 57 (13.4) 53 (6.6)	**(n, %)** 367 (86.6) 750 (93.4)	0.000
**Age in years (Mean ± S.D)**	54.7 ± 23.7	47.4 ± 26.1	0.005
**Patient type** - Inpatient - Outpatient	**(n, %)** 106 (8.9) 5 (10.6)	**(n, %)** 1075 (91.1) 42 (89.4)	0.643

**Table 10:** Pre-COVID-19 analysis of carbapenem resistance stratified by gender, age, and patient classification for *P. aeruginosa* (n = 413).

Variable	CP Resistant Strains (n = 115)	CP Sensitive Strains (n = 298)	*p*-Value
**Gender** - Male - Female	**(n, %)** 69 (27.6) 46 (28.2)	**(n, %)** 181(72.4) 117 (71.8)	0.891
**Age in years (Mean ± S.D)**	46.9 ± 25.9	49.3 ± 28.2	0.430
**Patient type** - Inpatient - Outpatient	**(n, %)** 111 (28.2) 4 (20.0)	**(n, %)** 282 (71.8) 16 (80.0)	0.422

**Table 11:** During-COVID-19 analysis of carbapenem resistance stratified by gender, age, and patient classification for *P. aeruginosa* (n = 644).

Variable	CP Resistant Strains (n = 128)	CP Sensitive Strains (n = 515)	*p*-Value
**Gender** - Male - Female	**(n, %)** 79 (22.3) 49 (17.0)	**(n, %)** 276 (77.7) 239 (83.0)	0.098
**Age in years (Mean ± S.D)**	55.0 ± 24.2	53.2 ± 27.0	0.491
**Patient type** - Inpatient - Outpatient	**(n, %)** 123 (19.6) 5 (27.8)	**(n, %)** 503 (80.4) 13 (72.2)	0.394

**Table 12:** Results of multinomial logistic regression analysis for predicting CP (carbapenem) resistance.

Variable	OR (95% CI)	*p*-Value
*E. coli* - with reference to *K. pneumoniae* - with reference to *P. aeruginosa*	0.06 (0.05–0.08) 0.03 (0.02–0.05)	0.000 0.000
*Klebsiella pneumoniae* - with reference to *E. coli* - with reference to *P. aeruginosa*	15.31 (11.62–20.14) 0.52 (0.50–0.63)	0.000 0.000
*Pseudomonas aeruginosa* - with reference to *E. coli* - with reference to *K. pneumoniae*	28.84 (21.62–38.20) 1.93 (1.66–2.34)	0.000 0.000

**Table 13:** Results of binary logistic regression analysis for predicting CP (carbapenem) resistance.

Variable	OR (95% CI)	*p*-Value
Male	2.50 (2.12–2.90)	0.000
Age	1.10 (1.04–1.12)	0.000
Inpatients	0.69 (0.46–1.05)	0.084
During COVID	0.43 (0.37–0.51)	0.000

## Data Availability

Data will be made available upon reasonable request directed to ibraheemaltamimi02@gmail.com.

## Abbreviations

AMR—antimicrobial resistance; CFU—colony forming units; CLSI—Clinical Laboratory Standard Institute; ESBL—extended spectrum beta-lactamase; UTI—urinary tract infection; CRE- carbapenem-resistant Enterobacterales; CPR- carbapenem resistance.

## References

[1] Stamm W.E., Norrby S.R. (2001). Urinary Tract Infections: Disease Panorama and Challenges. J. Infect. Dis..

[2] Flores-Mireles A., Hreha T.N., Hunstad D.A. (2019). Pathophysiology, Treatment, and Prevention of Catheter-Associated Urinary Tract Infection. Top. Spinal Cord Inj. Rehabil..

[3] Ku J.H., Bruxvoort K.J., Salas S.B., Varley C.D., Casey J.A., Raphael E., Robinson S.C., Nachman K.E., Lewin B.J., Contreras R. (2023). Multidrug Resistance of *Escherichia coli* from Outpatient Uncomplicated Urinary Tract Infections in a Large United States Integrated Healthcare Organization. Open Forum Infect. Dis..

[4] Sabih A., Leslie S.W. (2023). Complicated Urinary Tract Infections.

[5] Dai B., Liu Y., Jia J., Mei C. (2010). Long-Term Antibiotics for the Prevention of Recurrent Urinary Tract Infection in Children: A Systematic Review and Meta-Analysis. Arch. Dis. Child..

[6] Flores-Mireles A.L., Walker J.N., Caparon M., Hultgren S.J. (2015). Urinary Tract Infections: Epidemiology, Mechanisms of Infection and Treatment Options. Nat. Rev. Microbiol..

[7] Agegnehu A., Worku M., Nigussie D., Lulu B., Tadesse B.T. (2020). Pediatric Febrile Urinary Tract Infection Caused by ESBL Producing Enterobacteriaceae Species. BioMed Res. Int..

[8] Goyal D., Dean N., Neill S., Jones P., Dascomb K. (2019). Risk Factors for Community-Acquired Extended-Spectrum Beta-Lactamase–Producing Enterobacteriaceae Infections—A Retrospective Study of Symptomatic Urinary Tract Infections. Open Forum Infect. Dis..

[9] Tabasi M., Karam M.R.A., Habibi M., Mostafavi E., Bouzari S. (2016). Genotypic Characterization of Virulence Factors in *Escherichia coli* Isolated from Patients with Acute Cystitis, Pyelonephritis and Asymptomatic Bacteriuria. J. Clin. Diagn. Res..

[10] Pana Z.D., Zaoutis T. (2018). Treatment of Extended-Spectrum β-Lactamase-Producing (ESBLs) Infections: What Have We Learned until Now?. F1000Research.

[11] Kawamura K., Nagano N., Suzuki M., Wachino J., Kimura K., Arakawa Y. (2017). ESBL-Producing *Escherichia coli* and Its Rapid Rise among Healthy People. Food Saf..

[12] Vachvanichsanong P., McNeil E.B., Dissaneewate P. (2021). Extended-Spectrum Beta-Lactamase *Escherichia coli* and *Klebsiella pneumoniae* Urinary Tract Infections. Epidemiol. Infect..

[13] Abalkhail A., AlYami A.S., Alrashedi S.F., Almushayqih K.M., Alslamah T., Alsalamah Y.A., Elbehiry A. (2022). The Prevalence of Multidrug-Resistant *Escherichia coli* Producing ESBL among Male and Female Patients with Urinary Tract Infections in Riyadh Region, Saudi Arabia. Healthcare.

[14] De Kraker M.E.A., Stewardson A.J., Harbarth S. (2016). Will 10 Million People Die a Year Due to Antimicrobial Resistance by 2050?. PLoS Med..

[15] Ventola C.L. (2015). The Antibiotic Resistance Crisis: Part 1: Causes and Threats. Pharm. Ther..

[16] Knight G.M., Glover R.E., McQuaid C.F., Olaru I.D., Gallandat K., Leclerc Q.J., Fuller N.M., Willcocks S.J., Hasan R., van Kleef E. (2021). Antimicrobial Resistance and COVID-19: Intersections and Implications. eLife.

[17] Altamimi I., Almazyed A., Alshammary S., Altamimi A., Alhumimidi A., Alnutaifi R., Malhis M., Altamimi A. (2023). Bacterial Pathogens and Antimicrobial Susceptibility Patterns of Urinary Tract Infections in Children during COVID-19 2019–2020: A Large Tertiary Care Center in Saudi Arabia. Children.

[18] Mansouri F., Sheibani H., Javedani Masroor M., Afsharian M. (2019). Extended-Spectrum Beta-Lactamase (ESBL)-Producing *Enterobacteriaceae* and Urinary Tract Infections in Pregnant/Postpartum Women: A Systematic Review and Meta-Analysis. Int. J. Clin. Pract..

[19] Langford B.J., So M., Simeonova M., Leung V., Lo J., Kan T., Raybardhan S., Sapin M.E., Mponponsuo K., Farrell A. (2023). Antimicrobial Resistance in Patients with COVID-19: A Systematic Review and Meta-Analysis. Lancet Microbe.

[20] Lai C.C., Chen S.Y., Ko W.C., Hsueh P.R. (2021). Increased Antimicrobial Resistance during the COVID-19 Pandemic. Int. J. Antimicrob. Agents.

[21] Hassounah M., Raheel H., Alhefzi M. (2020). Digital Response during the COVID-19 Pandemic in Saudi Arabia. J. Med. Internet Res..

[22] Bono M.J., Leslie S.W., Reygaert W.C., Doerr C. (2021). Uncomplicated Urinary Tract Infections (Nursing).

[23] Giesen L.G., Cousins G., Dimitrov B.D., Van De Laar F.A., Fahey T. (2010). Predicting Acute Uncomplicated Urinary Tract Infection in Women: A Systematic Review of the Diagnostic Accuracy of Symptoms and Signs. BMC Fam. Pract..

[24] Hay A.D., Birnie K., Busby J., Delaney B., Downing H., Dudley J., Durbaba S., Fletcher M., Harman K., Hollingworth W. (2016). Microbiological Diagnosis of Urinary Tract Infection by NHS and Research Laboratories.

[25] Cornaglia G., Courcol R., Herrmann J.L., Kahlmeter G., Peigue-Lafeuille H., Jordi V. (2012). European Manual of Clinical Microbiology.

[26] Vandepitte J. (2003). Basic Laboratory Procedures in Clinical Bacteriology.

[27] Public Health England UK Standards for Microbiology Investigations. Investigation of urine. B 41 Issue 8.7. https://assets.publishing.service.gov.uk/government/uploads/system/uploads/attachment_data/file/770688/B_41i8.7.pdf.

[28] BioMérieux USA API® Reference Guide. https://www.biomerieux-usa.com/sites/subsidiary_us/files/18_api-ref-guide_v7.pdf.

[29] Carroll K.C., Glanz B.D., Borek A.P., Burger C., Bhally H.S., Henciak S., Flayhart D. (2006). Evaluation of the BD Phoenix Automated Microbiology System for Identification and Antimicrobial Susceptibility Testing of *Enterobacteriaceae*. J. Clin. Microbiol..

[30] BD PhoenixTM Panels—BD. https://www.bd.com/en-eu/offerings/capabilities/microbiology-solutions/clinical-microbiology/identification-and-susceptibility-testing/bd-phoenix-automated-identification-and-susceptibility-testing-system/bd-phoenix-panels.

[31] Kosikowska U., Andrzejczuk S., Grywalska E., Chwiejczak E., Winiarczyk S., Pietras-Ożga D., Stępień-Pyśniak D. (2020). Prevalence of Susceptibility Patterns of Opportunistic Bacteria in Line with CLSI or EUCAST among *Haemophilus parainfluenzae* Isolated from Respiratory Microbiota. Sci. Rep..

[32] Giske C.G. (2012). EUCAST Subcommitee for Detection of Resistance Mechanisms.

[33] Bradford P.A. (2001). Extended-Spectrum β-Lactamases in the 21st Century: Characterization, Epidemiology, and Detection of This Important Resistance Threat. Clin. Microbiol. Rev..

[34] WHO (2014). Antimicrobial Resistance.

[35] Karanika S., Karantanos T., Arvanitis M., Grigoras C., Mylonakis E. (2016). Fecal Colonization with Extended-Spectrum Beta-Lactamase–Producing *Enterobacteriaceae* and Risk Factors Among Healthy Individuals: A Systematic Review and Metaanalysis. Clin. Infect. Dis..

[36] Lemenand O., Coeffic T., Thibaut S., Colomb Cotinat M., Caillon J., Birgand G. (2021). Decreasing Proportion of Extended-Spectrum Beta-Lactamase among *E. coli* Infections during the COVID-19 Pandemic in France. J. Infect..

[37] Wardoyo E.H., Suardana I.W., Yasa I.W.P.S., Sukrama I.D.M. (2021). Antibiotics Susceptibility of *Escherichia coli* Isolates from Clinical Specimens before and during COVID-19 Pandemic. Iran. J. Microbiol..

[38] Nordmann P., Dortet L., Poirel L. (2012). Carbapenem Resistance in *Enterobacteriaceae*: Here Is the Storm!. Trends Mol. Med..

[39] Tacconelli E., Carrara E., Savoldi A., Harbarth S., Mendelson M., Monnet D.L., Pulcini C., Kahlmeter G., Kluytmans J., Carmeli Y. (2018). Discovery, Research, and Development of New Antibiotics: The WHO Priority List of Antibiotic-Resistant Bacteria and Tuberculosis. Lancet Infect. Dis..

[40] Adegoke A.A., Ikott W.E., Okoh A.I. (2022). Carbapenem Resistance Associated with Coliuria among Outpatient and Hospitalised Urology Patients. New Microbes New Infect..

[41] Mareș C., Petca R.C., Petca A., Popescu R.I., Jinga V. (2022). Does the COVID-19 Pandemic Modify the Antibiotic Resistance of Uropathogens in Female Patients? A New Storm?. Antibiotics.

[42] Taha R., Mowallad A., Mufti A., Althaqafi A., Fatani A.A., El-Hossary D., Ossenkopp J., AlhajHussein B., Kaaki M., Jawi N. (2023). Prevalence of Carbapenem-Resistant *Enterobacteriaceae* in Western Saudi Arabia and Increasing Trends in the Antimicrobial Resistance of *Enterobacteriaceae*. Cureus.

[43] Mareș C., Petca R.C., Popescu R.I., Petca A., Geavlete B.F., Jinga V. (2023). Uropathogens’ Antibiotic Resistance Evolution in a Female Population: A Sequential Multi-Year Comparative Analysis. Antibiotics.

